# Challenges to Student Interdisciplinary Learning Effectiveness: An Empirical Case Study

**DOI:** 10.3390/jintelligence10040088

**Published:** 2022-10-17

**Authors:** Cong Xu, Chih-Fu Wu, Dan-Dan Xu, Wen-Qian Lu, Kai-Yi Wang

**Affiliations:** 1 The Graduate Institute of Design Science, Tatung University, Taipei 104, Taiwan; 2 The Department of Industrial Design, Tatung University, Taipei 104, Taiwan

**Keywords:** challenges of interdisciplinary learning, learning outcomes, interdisciplinary integration ability, student attributes, learning environment

## Abstract

In order to meet industrial demands, some colleges and universities have offered interdisciplinary programs that integrate design, engineering, and business. However, how many changes these programs have brought to students, and whether students participating in these programs have had better interdisciplinary ability than students involved in a single discipline study have always been questions that many researchers want to explore. In a university that offers an interdisciplinary program, we found that there is no significant difference in interdisciplinary integration ability between the students participating in the interdisciplinary program and the students involved in a single discipline study through quantitative comparisons of 91 student questionnaires and analyses of interviews with nine teachers of interdisciplinary courses and other related staff members. This may result from the students’ lack of motivation, lack of prior experience, the influence of individual traits, the increase of learning pressure and academic burden, and the interference of disciplinary factors during interdisciplinary learning. The research finding is intended to improve student interdisciplinary learning effectiveness by facilitating interdisciplinary teachers’ understanding of the influencing factors of student interdisciplinary learning, and by providing a reference for interdisciplinary teaching design.

## 1. Introduction

In the past 20 years, discipline-based university education has undergone a great transformation towards interdisciplinary education. Interdisciplinary education and learning have become the focus of education and teaching research today (Klaassen 2018). There has been growing interest in interdisciplinary education and the publications of interdisciplinary education research have increased significantly (Heikkinen and Räisänen 2018). However, how effective is interdisciplinary education? To what extent have students improved their interdisciplinary ability? Although some studies in the fields of medical and nursing education have responded to these issues in recent years (Bullard et al. 2019; Liu 2021), there is obviously a lack of interest in research in the fields of engineering and computer science education (Heikkinen and Räisänen 2018). Overall, due to the challenges of effectiveness evaluation on interdisciplinary education, existing research has paid little attention to the growth and evaluation of students’ interdisciplinary ability (Lattuca et al. 2017a; Gao et al. 2020). It is even more difficult to find relevant literature on interdisciplinary teaching or empirical research (Lindvig and Ulriksen 2019; Van den Beemt et al. 2020). Therefore, we could hardly find studies that demonstrate the effectiveness of interdisciplinary education through teaching and learning practices in class (Gao et al. 2020). Are students involved in interdisciplinary education significantly different from students involved in a single discipline study in interdisciplinary ability? What specific teaching and learning factors affect the improvement of students’ interdisciplinary ability during interdisciplinary learning? Little has been learned so far. Biggs proposed a system model for teaching activities, which consists of four parts: student attributes, learning environment, learning process, and learning outcomes. Each part of the model follows the principle of alignment (Biggs 1993). Spelt et al. believed that this theoretical model could more comprehensively explain the interrelationships among various elements in interdisciplinary teaching and pointed out that “interdisciplinary integration ability” as an interdisciplinary learning outcome would be affected by “student attributes” and “interdisciplinary learning environment” (Spelt et al. 2009). This theory was applied and confirmed in subsequent studies by Spelt and Liu et al. (Spelt et al. 2015; Liu et al. 2022). In this paper, we will combine this theory and previous research to discuss the definition and connotation of interdisciplinary integration ability, as well as the factors affecting students’ learning outcomes in the area of interdisciplinary learning environment and student attributes.

### 1.1. Interdisciplinarity Integration Ability

**Figure 1:**
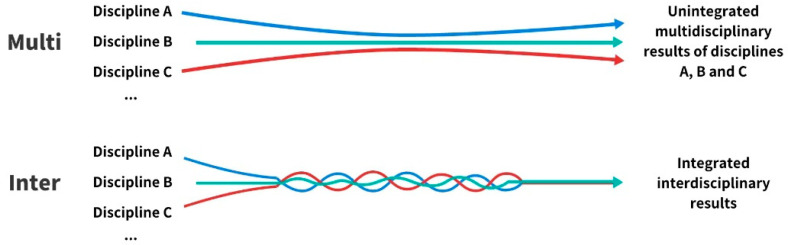
Difference between Multidisciplinarity and Interdisciplinarity. Collated, modified and drawn from Menken Steph, Keestra Machiel, Rutting Lucas, Post Ger, De Roo Mieke, Blad Sylvia, De Greef Linda. 2016. *An introduction to interdisciplinary research: Theory and practice.* Amsterdam: Amsterdam University Press, pp. 31–32.

Spelt et al. believed that interdisciplinary integration ability is also interdisciplinary thinking, including interdisciplinary knowledge and interdisciplinary skills (Spelt et al. 2009). The research findings of Menken et al. show that the key to interdisciplinarity being different from multidisciplinarity is the integration of related concepts, insights, theories and/or methods from different disciplines (Menken et al. 2016) (see Figure 1). Lattuca et al. argued that interdisciplinary ability enables students to integrate knowledge and methods from different domains for a comprehensive understanding of a problem (Lattuca et al. 2017a). Spelt et al. pointed out that the decisive feature of interdisciplinarity is the ability to integrate disciplinary knowledge. If there is no cultivation and training of this ability during teaching, but simply increased knowledge of different disciplines, it can still only be called multidisciplinary education (Spelt et al. 2015). Based on previous research and assertions, it is not difficult for us to come to the conclusion that interdisciplinary integration should be the core and key of interdisciplinarity. Therefore, the improvement of interdisciplinary integration ability should be the key to the evaluation of interdisciplinary teaching effectiveness and the concrete representation of students’ interdisciplinary learning outcomes. However, although “interdisciplinary integration ability” is of critical importance to interdisciplinary teaching, there is not yet a unified definition in the scientific and pedagogical literature (Danilova 2018), and the expressions of its connotation vary. For example, the IPEC (Interprofessional Education Collaborative) in the US defines core interdisciplinary ability as values/ethics for interprofessional practice, roles/responsibilities, interprofessional communication, and teams and teamwork. In addition, core interdisciplinary ability defined by the University of Virginia (2016) includes: communication, professionalism, shared problem-solving, shared decision making, and conflict resolution (Chen et al. 2017). Wilhelmsson et al. pointed out that interdisciplinary ability should include: teamwork and group processes, reflection and documentation, communication, shared knowledge or general common knowledge base, and ethics (Wilhelmsson et al. 2012). Interdisciplinary ability advocated by Lattuca et al. (2013) includes: awareness of disciplinarity, appreciation of disciplinary perspectives, appreciation of non-disciplinary perspectives, recognition of disciplinary limitations, interdisciplinary evaluation, ability to find common ground, reflexivity, and integrative skill; and subsequently these eight abilities are extracted into three: interdisciplinary skill, reflective behavior, and recognizing disciplinary perspectives (Lattuca et al. 2013). The definitions of the above-mentioned core interdisciplinary integration ability are slightly different, but they all highlight similar abilities (see Table 1), including: interdisciplinary communication, facilitating the formation of shared knowledge base and problem-solving teamwork, interdisciplinary reflection and evaluation, accepting other disciplinary values or perspectives, having disciplinary awareness and perspectives, being able to recognize disciplinary limitations, integrating knowledge from different disciplines to deal with complex problems, professionalism, and other skills.

### 1.2. Student Attributes

Student attributes include motivation, individual traits, prior experience, etc. (Spelt et al. 2009, 2015; Liu et al. 2022). Some researchers discussed the motivations and goals of interdisciplinary learners. For example, Barnard pointed out that most students generally hold conflicting views on interdisciplinary learning (Barnard et al. 2013). In their research, Kabo et al. mentioned that some reports indicated that people with engineering educational background put up resistance to interdisciplinary learning goals (Kabo and Baillie 2009). Berasategi et al. believed that student individual conditions, including learning motivation and maturity, are closely related to their development of interdisciplinary thinking (Berasategi et al. 2020). Some scholars discussed that learners’ prior experience seems to have an impact on interdisciplinary learning outcomes. Heiman pointed out that freshman students are reluctant to use learning methods different from what they have adopted in high school (Heiman 2014). Studies found that students’ prior learning experience of a single discipline makes them feel overwhelmed and at a loss when faced with the teaching design and expectations of interdisciplinary courses (Strain and Potter 2012).

### 1.3. Interdisciplinary Learning Environment

Interdisciplinary learning environment includes elements like courses, teachers, pedagogy, assessment, etc. (Spelt et al. 2009, 2015; Liu et al. 2022). Van den Beemt et al. suggested that any teachers and students involved in interdisciplinary education projects should be aware of the relation among the specific perspectives and visions of interdisciplinary education, and the chosen teaching methods (Van den Beemt et al. 2020). Do believed that interdisciplinary courses should have a goal that can be achieved within a semester, while corresponding tasks should be designed and learning objectives should be set related to the level of difficulty (Do 2013). Chen et al. pointed out that a course study load is critical to the effectiveness of interdisciplinary learning (Chen et al. 2009). Hansen et al. believed the motivations and goals of the teaching program as the basis for an interdisciplinary approach to pedagogy in the context of interdisciplinary curriculum development (Hansen and Dohn 2017). For the setting of teaching content, Biggs emphasized that if students want to obtain the desired learning outcomes, the basic task of teachers is to engage students in learning activities that may lead them to achieve these outcomes; during the process, deciding what students learn is far more important than what teachers do (Biggs 1993). In addition, many scholars discussed teaching activities, curriculum design, teachers, teaching methods, assessment and other topics in terms of interdisciplinary learning environment (Carreras Marín et al. 2013; Gómez Puente et al. 2013; Gouvea et al. 2013; Jones 2010; Lindvig and Ulriksen 2019).

The literature review provides a research framework for us to explore the effectiveness of interdisciplinary curriculum teaching practice that aims at the cultivation of interdisciplinary integration ability, and the influencing factors. Meanwhile, in view of the relative lack of empirical reports on interdisciplinary teaching, this paper will try to find out whether the students participating in an interdisciplinary program have a significant advantage over the students involved in a single discipline study by comparing their interdisciplinary integration ability through an empirical case study. Moreover, this paper will further analyze which elements in the areas of “student attributes” and “interdisciplinary learning environment” affect the interdisciplinary integration ability of the students participating in the interdisciplinary program based on the collected sample data and materials.

## 2. Materials and Methods

### 2.1. Setting and Teaching of Interdisciplinary Courses

In the face of rapid technological change, global climate change, and an ever-changing market, product innovation and sustainable development of manufacturing are no longer complex problems that can be completely solved by a single discipline. Some scholars pointed out that the life cycle of a product is divided into three stages: design, engineering, and sales, but these three stages are not independent, and on the contrary, they should be integrated (Buxton 2010). The researchers of design education indicated that as industrial projects are becoming increasingly complex and larger in scale, the boundaries between artifacts, structures, and processes are beginning to be more blurred. Since the requirements at each level are rising, the complexity of design problems will be significantly increased. Therefore, designers will be required to be familiar with working in interdisciplinary teams that integrate engineering and business (McDermott et al. 2014). In addition, many successful large international companies, such as GE, Sony, Philip, etc., have adopted the design perspective as a problem-solving tool for the entire company and a key element in the formation of corporate strategies. Design is increasingly recognized as a key to the success of business practices, and design thinking has become increasingly popular in the field of business (Matthews and Wrigley 2017). Gill et al. believed that interdisciplinarity integrating mechanics, electronics, information technology and design is the future of Industry 4.0, and the integration of these majors will create solutions for complex problems faced by intelligent manufacturing, and product innovation and development (Gill et al. 2021). Driven by industrial development, in fact, some educational institutions have begun to try to carry out interdisciplinary teaching activities that integrate design, business, and engineering technology, such as Jiangnan University in mainland China, Arizona State University in the United States, etc. (McDermott et al. 2014; Li et al. 2019).

#### 2.1.1. Setting of Interdisciplinary Courses

The interdisciplinary courses mentioned in this paper are developed and designed by a comprehensive university in Taiwan according to the above-mentioned industrial talent development trend. The university’s mission is to cultivate applied and compound talent for industrial development. A great number of leaders of large international enterprises have graduated from the school successively. Every year, the school regularly invites people from the industry, including prominent alumni, to discuss with the school’s teachers and educational administrators industrial talent needs as well as current education trends and issues. The school’s interdisciplinary program was established in this context. The program is aimed at students majoring in Industrial Design, Media Design, Materials Engineering, Mechanical Engineering, Electrical Engineering, Computer Science and Engineering, Business Management, Applied Foreign Languages, etc. It integrates courses and teaching resources in the fields of business, engineering and design, with the goal of cultivating interdisciplinary integration ability, to form an interdisciplinary curriculum system consisting of interdisciplinary basic courses (i.e., introductory courses for business, engineering, and design majors) + interdisciplinary integration courses like Capstone + internship and practical courses. The interdisciplinary program is intended to improve students’ skills in interdisciplinary communication, interdisciplinary teamwork, interdisciplinary reflection and evaluation, interdisciplinary values or viewpoints, disciplinary limitation cognition, and interdisciplinary knowledge integration.

This program offers 23 courses, including Business Analysis, Applied Electronic Creation, Materials Processing and Analysis, Design Fundamentals, Capstone, etc. These courses are arranged in various stages from the first year to the fourth year in this university, and there is a progressive relationship between the courses before and after (see Table 2 for details). This is a semi-closed academic program, exit only and no entry. Therefore, students must join this program in the first semester of their freshman year and those who try to join the program midway are rejected. In addition to completing the courses of their own majors, they need to complete the various courses of the interdisciplinary program. Meanwhile, students can be exempted from taking the interdisciplinary basic courses in this program within their own disciplines. In addition, after the start of the program, the courses that have been registered and selected during the semester cannot be withdrawn, but in accordance with the principle of voluntariness, the participants are allowed to stop the study of subsequent unselected courses and withdraw from the program. 

#### 2.1.2. Teaching of Interdisciplinary Courses

More than 30 teachers from the College of Engineering, College of Management, and College of Design are involved in this interdisciplinary program. The interdisciplinary basic courses are taught by the teachers from these three colleges respectively. Since most of these courses belong to introductory or entry level courses, the teachers of each college mainly adopt the teaching method that combines more traditional theoretical lectures and practice training to cultivate students’ cognition of other disciplines’ values, viewpoints and methods. The students participating in these courses are not students of the disciplines to which the courses belong. The teaching of Capstone interdisciplinary integration courses is led and aided by an interdisciplinary teaching team composed of teachers from the three colleges in PBL teaching mode. All students participating in these interdisciplinary capstones are required to form interdisciplinary teams when taking these courses to complete corresponding subject training by solving real and complex problems.

### 2.2. Measurements and Interviews

Self-assessment comparisons of interdisciplinary integration ability indicators were made in the above-mentioned university between the students in higher grades participating in an interdisciplinary program and the students in higher grades involved in a single discipline study. Considering that there may be some subjective bias of student self-assessments, the research group planned to conduct sample interviews with the tested students to more objectively evaluate the difference in interdisciplinary integration ability of this group of students by collecting their coursework and referring to expert evaluations. However, due to some obstacles, such as the students’ unwillingness and earlier departure of graduates caused by temporary school closures during the COVID-19 pandemic, the research group failed to collect relevant students’ interview materials and interdisciplinary coursework. Therefore, members of the research group interviewed the teachers, tutors, and program administrators involved in interdisciplinary teaching in the university, to ask about their understanding of these students. It is intended to more objectively understand the performance of this group of students making self-assessments from the perspectives of the teachers. All the students participated in the self-assessments of interdisciplinary integration ability indicators voluntarily, and all the staff members invited to the interviews approved the acquisition and study of the interview recordings. The details are as follows:

#### 2.2.1. Interdisciplinary Integration Ability Measurements

Chen, Wang et al. synthesized previous research findings, summarized the connotations of interdisciplinary integration ability discussed in the first part of this paper and defined it as “common interdisciplinary integration-based core competencies”, i.e., shared interdisciplinary integration ability that students of different disciplines should have. Based on this definition and combining the characteristics of the cultural background and native language habits of local students growing up and the general conditions of curriculum teaching, they developed an interdisciplinary integration ability scale (see Appendix A for details), which specifically includes three sub-ability measurements, i.e., “interdisciplinary communication, interdisciplinary reflection, and interdisciplinary practice”, and a total of 16 questions (Chen et al. 2017). Among them, “interdisciplinary communication” is reflected in the three main indicators of “respecting professional opinions, understanding different professional terms, and communicating through communication tools”, and includes six questions; “interdisciplinary reflection” is reflected in the three main indicators of “understanding the role differences among people with different expertise, making a reflection and generating new ideas through the process of interaction with others, and reflecting on the problems encountered in the process”, and includes five questions; “interdisciplinary practice” is reflected in the three main indicators of “discovering teamwork problems and proposing practical solutions, evaluating the work efficiency of team members, and evaluating the effectiveness and making suggestions for improvement”, and includes five questions. The 16 questions are answered based on a 5-point Likert scale, where 1 is strongly disagree, 2 is somewhat disagree, 3 is neutral/no opinion, 4 is somewhat agree, and 5 is strongly agree. This scale has been used in universities in Taiwan and has shown good reliability. In this case, considering the school’s understanding of the cultivation of students’ interdisciplinary integration ability, after discussing with some research experts, the research group adopted this scale to make measurement comparisons of “interdisciplinary communication, interdisciplinary reflection, and interdisciplinary practice” between the students participating in the interdisciplinary program and the students involved in a single discipline study in the university. In addition, in the pretests before using the scale, a total of 60 questionnaires were distributed, and 60 valid questionnaires were collected. The reliability value of Cronbach’s α is .88, which indicates high reliability of the questionnaire design and thus it can be used for testing. 

Samples of Students Participating in the Interdisciplinary Program

The students participating in the interdisciplinary program are undergraduate students in higher grades majoring in Industrial Design, Media Design, Materials Engineering, Mechanical Engineering, Electrical Engineering, Computer Science and Engineering, and Business Management from the above-mentioned university. When they entered the university in the first year, they attended the introduction meeting for the interdisciplinary program, and signed up to participate in the program. The research group conducted a random sampling of the students in higher grades who have completed the integrated Capstone courses, and collected 19 valid interdisciplinary student samples (excluding the student samples for the pretests), including three samples of Industrial Design majors, four samples of Media Design majors, five samples of Materials Engineering majors, three samples of Mechanical Engineering majors, one sample of Electrical Engineering major, and three samples of Computer Science and Engineering majors.

Samples of Students not Participating in the Interdisciplinary Program

The research group randomly distributed questionnaires to the students in higher grades in the university who have not participated in the interdisciplinary program. A total of 72 valid questionnaires were collected (excluding the questionnaires for the pretests), and among them, 23 are from Industrial Design majors, 29 are from Materials Engineering majors, and 20 are from Electrical Engineering majors.

For details of the independent variables, dependent variables and the number of students tested in the measurement comparisons, please refer to Table 3.

In addition, it should be noted in this paper that, due to factors such as students’ unwillingness, the research group were not able to collect any questionnaires from the students majoring in Business Management whether they participated in the interdisciplinary program or not.

#### 2.2.2. Interview 

Interview Design

The purpose of these interviews is to verify whether the teachers’ observations of the students participating in the interdisciplinary program are consistent with the students’ self-assessments of their own interdisciplinary integration ability. In this way, the research group can more objectively understand the differences in learning outcomes and learning status between the interdisciplinary students and non-interdisciplinary students of various majors and discuss what factors may affect the improvement of student interdisciplinary integration ability based on these differences. In order to more comprehensively grasp the learning conditions of the students participating in the interdisciplinary program, the research group decided to conduct focus group interviews. When considering the focuses of these interviews, in order to avoid the situation that the respondents may be induced by the vocabulary with a specific connotation, the research group did not place related words mentioned above such as students’ “learning methods, expectations, individual traits, prior experience, learning motivation, and maturity” in the questions when designing the discussion guide. An open-ended interview structure has been introduced to avoid any leading questions or guided answers. The clues are as follows:

Q1. What is the classroom atmosphere during class?

Q2. What is the performance of students in course learning and task completion? Are there any significant differences among them in this regard? Are the students of various majors the same in this regard?

Q3. When the courses are over, can the students achieve the ability target set when the courses were opened? If not, what is the reason? Are the students of various majors the same in this regard?

Interview Process

A total of 9 staff members (Pt-1 to Pt-9) from Industrial Design, Economics, Materials Engineering, Mechanical Engineering, and other disciplines were invited to the interviews. They are teachers of the interdisciplinary program, tutors of the interdisciplinary students, and program and teaching administrators. The respondents were asked to answer questions and have discussions according to the above clues based on their knowledge of the students in higher grades participating in the interdisciplinary program. These interviews were open-ended, and all the respondents fully expressed their opinions without any pressure or inducement. A total of 9 respondents have been interviewed, with the full process of interviews completed in 4 separated durations. The interviews were recorded only upon the agreement by the respondents. A total of 5 h, 49 min, and 13 s of audio recordings were produced.

Interview Analytical Methods

All the interview recordings were transcribed verbatim. Questions, explanations of questions, and follow-up questions in the verbatim transcript were removed, followed by a coding analysis of the verbatim transcript. The coding analysis was made within the theoretical framework of interdisciplinary integration ability, student attributes, and interdisciplinary learning environment discussed in the first part of this paper. After open coding, the textual material produced 185 units of thematic encoding distributed over 86 nodes. The same unit of thematic coding may belong to different nodes, but the same node can only be categorized into one subcategory and cannot be categorized into another subcategory. Therefore, through further summarization, mergence, and sorting, 18 subcategories were formed, and 8 categories were finally extracted. Due to the failure of sample collection of student coursework that can directly reflect students’ interdisciplinary ability, we could only rely on teachers’ judgments and subjective evaluations to understand students’ interdisciplinary learning and to infer the improvement of their interdisciplinary integration ability. Therefore, the research group used the three dimensions of “learning condition feedback, student attributes, and learning environment” to carry out axial coding of the 8 categories, instead of “interdisciplinary integration ability, student attributes, and learning environment”.

To ensure the high reliability of the initial open coding, the research assistants randomly selected 20% of the interview data for coding and compared it with the previous coding of the same part, finding no significant difference.

## 3. Results

### 3.1. Self-Assessment Results of Student Core Interdisciplinary Integration Ability

This study compares the core interdisciplinary integration ability among the students in the Departments of Industrial Design, Materials, and Electrical Engineering who have not participated in the university’s interdisciplinary program and the students who have participated in the program. Questionnaires were distributed to the students in the Departments of Industrial Design, Electrical Engineering, and Materials, and interdisciplinary students, and a total of 91 valid questionnaires were collected. Among them, 23 are from Industrial Design students, 29 are from Materials students, 20 are from Electrical Engineering students, and 19 are from interdisciplinary students (including three from Industrial Design majors, four from Media Design majors, five from Materials Engineering majors, three from Mechanical Engineering majors, one from Electrical Engineering major, and three from Computer Science and Engineering majors). After sorting out the questionnaire data of the four different groups of students, the quantitative statistical method of one-way ANOVA was used for analysis. Please refer to Table 4 for the results.

The quantitative analysis results of the students in the three sub-ability measurements of communication, reflection, and practice (see Table 4) show that a total of five questions indicate significant differences: Interdisciplinary Communication 2, Interdisciplinary Communication 5, Interdisciplinary Reflection 1, Interdisciplinary Reflection 3, and Interdisciplinary Practice 1. This means that each of the five questions can show significant differences in core interdisciplinary integration ability among the students of at least two or more disciplines. Post hoc multiple comparisons were required to explore specific situations. In addition, the results of the test of homogeneity of variances (see Table 5) show that Interdisciplinary Communication 3, Reflection 4, Practice 4 and Practice 5 all have the characteristics of heterogeneity of variance, which indicates the distribution of the samples of the above four questions, i.e., the degree of dispersion is very significantly heterogeneous. Therefore, in the case of multiple comparisons, these four questions needed to be tested by the Games–Howell method instead of the Scheffe method (Mamiseishvili et al. 2016). The results of the multiple comparisons indicate that although there are significant differences in the previous One-way ANOVA, Communication 2, Reflection 3, and Practice 1 do not show any significant differences in multiple comparisons, and finally only Interdisciplinary Communication 5, Interdisciplinary Reflection 1, and Interdisciplinary Practice 4 show significant differences (see Table 6 for details). The specific findings are as follows: the scores of Industrial Design students and Materials Engineering students in Communication 5 are significantly higher than those of Electrical Engineering students, and their means show the relation of “Industrial Design students > Materials students > interdisciplinary students > Electrical Engineering students”, but the first three student groups do not show any significant difference, and there is no significant difference between interdisciplinary students and Electrical Engineering students. In Reflection 1, the means of the four student groups in core interdisciplinary integration ability show the relation of “Industrial Design students > interdisciplinary students > Materials students > Electrical Engineering students”, in which only the scores of Industrial Design students are significantly higher than those of Electrical Engineering students. There is no other significant difference in Reflection 1, and in other words, interdisciplinary students and Materials Engineering students are not significantly different from Industrial Design students or Electrical Engineering students, and meanwhile, there is no significant difference between interdisciplinary students and Materials Engineering students either. Practice 4 reflects the same situation as Reflection 1: the means of the four student groups in core interdisciplinary integration ability show the relation of “Industrial Design students > interdisciplinary students > Materials students > Electrical Engineering students”, and only the scores of Industrial Design students are significantly higher than those of Electrical Engineering students, and there is no significant difference among other student groups.

Students in the four groups show significant differences in only three out of the 16 questions of core interdisciplinary integration ability, and in these three questions, interdisciplinary students are not significantly different from any other student groups, which prevents us from drawing the conclusion that interdisciplinary students are significantly better than other students in core interdisciplinary integration ability. This result is surprising. Why the students participating in the interdisciplinary program do not have outstanding related ability deserves further discussions by the research group.

### 3.2. Qualitative Analysis Results of Teacher Interviews

Table 7 shows how many times the codes covering three dimensions (D-01 to D-03) were mentioned and how many teachers mentioned them. Among them, “Learning Condition Feedback” (D-01) was mentioned 36 times by six teachers in total, and it includes two categories (D-01-01 to D-01-02) and five subcategories (D-01-01a to D-01-02c); “Interdisciplinary Learning Environment” was mentioned 41 times by seven teachers in total, and it includes three categories (D-02-01 to D-02-03) and six subcategories (D-02-01a to D-02-03b); “Student Attributes” was mentioned 108 times by eight teachers in total, and it includes three categories (D-03-01 to D-03-03) and seven subcategories (D-03-01a to D-03-03b).

In order to find shared feelings of the respondents, we only analyzed subcategories mentioned by two or more respondents. There are 15 subcategories (D-01-01a, D-01-02a to D-01-02c, D-02-01a to D-02-02b, D-03-01a to D-03-03b) and seven categories (D-01-01, D-01-02, D-02-01, D-02-02, D-03-01 to D-03-03) involved. The frequencies of the three main axis dimensions and seven categories are shown in Table 8. The specifics of each main axis dimension will be explained in order.

#### 3.2.1. D-01 Learning Condition Feedback

This dimension includes the description and evaluation made by the teachers being interviewed on the behavioral performance of the interdisciplinary integration ability of the students participating in the interdisciplinary program. In the dimension, the teachers gave specific feedback on students’ interdisciplinary values, interdisciplinary knowledge integration, interdisciplinary teamwork, interdisciplinary communication, and interdisciplinary team consensus building, as well as students’ words, deeds, and emotions under the program. This dimension, including the two categories of positive feedback and negative feedback identified by their positive and negative connotations, was mentioned 36 times in total by six respondents successively.

In the category of “D-01-01 Positive Feedback”, only the subcategory of “D-01-01a” was mentioned by two respondents. It mainly records the two teachers’ recognition of the growth of some students in interdisciplinary learning, including overcoming the difficulties in interdisciplinary communication, reaching an interdisciplinary team consensus, and being willing to carry out interdisciplinary teamwork practice. They clearly stated that they have seen the students’ growth in interdisciplinary ability (see Appendix B). We therefore named D-01-01a “Growth in Interdisciplinary Integration Ability”. Unfortunately, positive feedback is the least among the seven categories in terms of the number of respondents who mention it and the frequency of mentions.

The category of “D-01-02 Negative Feedback” includes three subcategories: “D-01-02a, D-01-02b, and D-01-02c”. Six out of the nine respondents talked about the problems and negative words, deeds and emotions of students in higher grades in interdisciplinary learning from different perspectives (see Appendix B). D-01-02a mainly reflects that the students still do not understand or agree with interdisciplinary values after participating in the interdisciplinary program; some students have interdisciplinary communication barriers and need to rely on their teachers to interpret and explain interdisciplinary knowledge. These all reflect that the students are still far from the acquisition of interdisciplinary integration ability. Thus, we named D-01-02a “Problems with Interdisciplinary Ability”. D-01-02b was mentioned by two teachers, and it mainly describes students’ doubts or distrusts during interdisciplinary learning. They not only distrust interdisciplinary learning, but also have no confidence in teachers of other disciplines and in this program. We can understand that the students cannot accept the views and values of other disciplines, but we are very surprised to find that teachers of other disciplines and even the program itself cannot be trusted. As a result, we also listed this subcategory and named it “Student Distrust” instead of classifying it into the subcategory of “Problems with Interdisciplinary Ability”. A total of three teachers mentioned the subcategory of D-01-02c, and they mainly talked about students’ frustration, emotional ventilation, and withdrawal from the interdisciplinary program due to the intensity of the courses, the gap between expectations and perceived reality, and problems with teamwork. These are indeed negative phenomena that students experience during interdisciplinary learning, so we named this subcategory “Negative Emotions and Behaviors”.

Based on the above analyses, negative feedback was mentioned 28 times by six respondents, which is significantly more than the positive feedback. Within the dimension of “Learning Condition Feedback”, the frequency proportion of negative feedback accounts for 77.8%, which is more than three times that of positive feedback. From the analysis of this feedback, we believe that the overall improvement of students’ interdisciplinary integration ability under this program is not satisfactory.

#### 3.2.2. D-02 Interdisciplinary Learning Environment

The dimension obtained after the axial coding was mentioned 41 times by seven respondents. Two categories of “D-02-01” and “D-02-02” were mentioned by more than two respondents. In this dimension, the respondents described students’ feedback on the difficulty of interdisciplinary courses, the learning pressure imposed by the interdisciplinary teachers, the increased learning burden of interdisciplinary courses, and the influence of non-interdisciplinary teachers on the students participating in the interdisciplinary courses. Based on the previous discussion on the literature of “interdisciplinary learning environment”, we coded this dimension as “Interdisciplinary Learning Environment”.

The subcategories of D-02-01 include: D-02-01a and D-02-01b. D-02-01a was mentioned eight times by four respondents based on all the written materials of this study. The interviewed teachers described that the students reported that the basic design courses under this interdisciplinary program feature high-intensity learning, the engineering courses are too difficult to understand, so that they felt huge learning pressure, resulting in the negative emotions or behaviors mentioned above (see Appendix C for details). Hence, we named this sub-category “Study Pressure”. D-02-01b was mentioned 19 times by six respondents. The interdisciplinary teachers found that after a period of interdisciplinary learning, some students think that they have consumed too much time or energy in interdisciplinary learning instead of in their own discipline; and they worried that the final scores of the interdisciplinary courses will lower their average score, etc., all of which make students perceive interdisciplinary learning as a burden to their own discipline study (see Appendix C). Based on this, we named the subcategory “Academic Burden”, and the category of D-02-01 including the two subcategories of “D-02-01a Study Pressure and D-02-01b Academic Burden” “Pressure and Burden”.

The subcategories of D-02-02 include: D-02-02a and D-02-02b. D-02-02a was mentioned 10 times by three teachers. The respondents mainly mentioned the influence of teachers of mono-disciplines on students’ disciplinary thinking, which mostly features contempt or rejection of interdisciplinary values (see Appendix C). This seems to challenge students’ interdisciplinary values, and indeed affects students’ cognition, judgment and persistence in interdisciplinary learning to a considerable extent. Therefore, this subcategory was named “Influence of Departments”. D-02-02b was mentioned five times by five respondents. In this subcategory, the respondents mainly mentioned that when the students are switching between interdisciplinary courses and disciplinary courses under this program, the teachers of different disciplines have different evaluation criteria for the output of the same student, which can bring frustration and value conflicts to the students participating in the interdisciplinary program. Some students cannot adapt to, identify with, or accept different judging standards (see Appendix C). Therefore, D-02-02b was named “Differences among Disciplines”. 

Among the three main axis dimensions, interdisciplinary learning environment has the second highest number of respondents who mentioned it and the frequency of mentions. Two categories of D02-01 and D02-02 were both mentioned by six respondents. The former has a frequency of 19, and the frequency proportion within this dimension accounts for 46.3%; the latter has a frequency of 15, and the frequency proportion within this dimension accounts for 36.6%. In this dimension, the frequency difference between the two categories is about 10%. Based on this, we believe that the impact of interdisciplinary students’ learning pressure and burden may bring the respondents a stronger feeling than that of differences between disciplines. However, in any case, the interviews reveal such a finding, i.e., the intensity, pressure, and burden felt by the students in interdisciplinary learning, the unsupportive teachers of their own disciplines against interdisciplinary learning, and differences among disciplines are closely related to “D01-02 Negative Feedback” mentioned in the previous axis dimension.

#### 3.2.3. D-03 Student Attributes

This dimension records the judgments made by the interviewed teachers on the students’ learning motivation, and the speculation and description of the motivational causes after observing the students’ interdisciplinary learning status. It also includes a description of the impact of students’ experiences before being involved in the interdisciplinary program on their interdisciplinary learning, and the impact of students’ individual traits, such as personal characteristics, learning habits, and learning responsibility on their interdisciplinary learning. According to the discussion of “student attributes” in the first part of this paper, we named this dimension “Student Attributes”. The number of respondents who mentioned the dimension is the most and the frequency of mentions is the highest among the three dimensions. The topic of student attributes was mentioned 108 times in total by eight respondents successively. This dimension includes three categories: “D03-01, D03-02, and D03-03”.

In this dimension, the respondents talked about the phenomena of students lacking motivation, dawdling their time away, and being unwilling to participate in interdisciplinary learning, and believed that students lack interdisciplinary learning motivation. Meanwhile, the teachers made some interpretations and analyses of the reasons for the lack of motivation of the students (see Appendix D). Therefore, we named D03-01 “Motivation”. The number of respondents who mentioned D03-01 Motivation is the most and the frequency of mentions is the highest among the three categories. It was mentioned 56 times by eight respondents. The category of D03-01 Motivation includes three subcategories: “D03-01a, D03-01b, and D03-01c”. Self-determination theory suggests that pure curiosity or a desire to master can be called intrinsic motivation; all the other behaviors are driven by extrinsic motivation, derived from the integration and internalization of social values or rules (Cook and Artino 2016). Based on this, we classified the phenomena of the students’ lacking motivation, dawdling their time away, and being unwilling to participate in interdisciplinary learning, as well as other related phenomena of a lack of motivation into the subcategory of D03-01a, and named it “Intrinsic Motivation”. D03-01a was mentioned 38 times by eight respondents successively. In addition, two respondents mentioned the incentives or restraints that are intended to stimulate students’ extrinsic motivations and suggested that these extrinsic motivations can be transformed into students’ intrinsic motivations. This was encoded into D03-01b and named “Extrinsic Motivation”. The reasons for the lack of motivation mentioned by the teachers, including students’ identification with the teachers, whether interdisciplinary learning can meet their short-term realistic goals, and interdisciplinary learning’s relevance to their own disciplines were encoded into D03-01c and named “Source of Motivation”. The subcategory of D03-01c was mentioned by six respondents.

In the category of D03-02, the respondents talked about the students’ incompatibility with the teaching methods not belonging to their own discipline, and their unaccustomedness and irritation of PBL teaching when taking Capstone courses due to their lack of prior interdisciplinary learning experience. In addition, the students do not have similar experiences in social cognition and life practice before participating in interdisciplinary learning, especially before taking the interdisciplinary integration courses in the upper grades. In fact, they showed confusion about interdisciplinary cognition both before and after participating in the program (see Appendix D). The research team named this coding category “Prior Experience”. Meanwhile, the text content about interdisciplinary learning incompatibility due to the lack of prior learning experience was coded as the subcategory of “D-03-02a” and named “Influence of Prior Teaching and Learning Styles”; the relevant text content about the lack of interdisciplinary cognition in previous social cognition and life practice was coded as the subcategory of “D-03-02b”, and named “Prior Interdisciplinary Practice Experience and Cognition”. The number of respondents who mentioned D03-02 Prior Experience is the second most and the frequency of mentions is the second highest among the three categories. It was mentioned 29 times by seven respondents. D-03-02a was mentioned 21 times by six respondents. D-03-02b was mentioned 8 times by three respondents. The students from the Department of Design and the students from the Department of Engineering were compared, and it can be seen that different prior teaching styles of the two disciplines lead to different adaption conditions of the students after participating in the interdisciplinary program. For example, design students already have problem-oriented learning experience when they study in their own disciplines. Meanwhile, due to the nature of design disciplines, design students have more opportunities to be exposed to some interdisciplinary knowledge. As a result, design students are more adaptable in interdisciplinary learning, while students in other disciplines are the opposite. The teachers believed that the students’ lack of interdisciplinary experience and cognition before participating in the interdisciplinary program influences the formation of their interdisciplinary values or awareness, which may be one of the factors that cause the students to be at a loss or even withdraw from the program when facing interdisciplinary learning.

In the category of D03-03, the respondents reflected different characteristics of students in different departments in interdisciplinary learning; according to the content of the relevant text, the research group coded it as “D-03-03a” and named it “Different Characteristics of Students in Different Departments”. Besides, we coded the content about the impact of student personal characteristics like sense of responsibility, learning attitudes on interdisciplinary learning as D-03-03b, and named it “Student Personal Characteristics”. For the category of D03-03 that includes D-03-03a and D-03-03b, we named it “Individual Traits” (see Appendix D). Although the number of respondents who mentioned D03-03 and the frequency of mentions are the least among the three categories of the dimension of Student Attributes, it was mentioned 23 times by five respondents. D-03-03a was mentioned 10 times by three respondents. D-03-03b was mentioned 13 times by five respondents. From the encoded text, some interdisciplinary learning conditions of Engineering, Design, and Business students can be seen, and it is found that different student characteristics shaped by different discipline education also seem to have an impact on interdisciplinary learning. For example, Engineering students are relatively not good at communication, while Design students are more creative, and Business students are considered to be more inclined to take shortcuts in interdisciplinary learning. From the text of the encoded unit, it can be seen that the teachers felt that the students’ own personal characteristics, learning attitudes, and sense of responsibility can also have an impact on interdisciplinary learning (see Appendix D).

As mentioned above, the number of respondents who mentioned the dimension of Student Attributes is the most and the frequency of mentions is the highest among the three main axis dimensions. The frequencies of the three categories of “D03-01 Motivation, D03-02 Prior Experience, and D03-03 Individual Traits” are respectively 51.9%, 26.9%, and 21.3%. Among all the categories, the frequency proportion of Motivation accounts for 30.3%, the frequency proportion of Prior Experience accounts for 15.7%, and the frequency proportion of Individual Traits accounts for 12.4%. According to the interview transcript, the space of the dimension of Student Attributes is the greatest. It can be said that student attributes should have a very important relation with interdisciplinary learning outcomes, and they can play a key role in student interdisciplinary integration ability.

## 4. Discussion

The purpose of this study is to empirically explore whether there is significant difference in interdisciplinary integration ability between the undergraduate students participating in the interdisciplinary program that integrates design, engineering, and business, and the students studying a single discipline, and to discuss the reasons for the differences. To this end, the research group invited 91 students for self-assessment analyses of core interdisciplinary integration ability and nine teachers and related staff members involved in the interdisciplinary program to interviews on the conditions of the group of interdisciplinary students. The experimental data were obtained through quantitative comparative analyses and qualitative coding analyses. The results of quantitative analyses show that the students participating in the interdisciplinary program are not significantly different from those of other disciplines in the ability level of “interdisciplinary communication, interdisciplinary reflection, and interdisciplinary practice”. The results of qualitative analyses show that the teachers’ negative feedback on the interdisciplinary students is significantly more than positive feedback in the number of respondents who mentioned it and frequency of mentions. Meanwhile, through qualitative analyses, it is found that the interdisciplinary students’ disagreement with interdisciplinary values, distrust of interdisciplinary teachers, obstacles in interdisciplinary communication, and problems with teamwork. Therefore, the research team believes that the improvement of the students in ability after participating in interdisciplinary learning is not ideal. This clearly echo their insignificant interdisciplinary integration ability in the interdisciplinary integration ability measurements. Based on the results of the data analyses, we believe that the results of the qualitative analyses can confirm the objectivity of the students’ self-assessment results to a considerable extent. Based on this finding, we are more inclined to assert that there is no necessarily significant difference in interdisciplinary integration ability between the students participating in the interdisciplinary program and the students studying a single discipline. This may not support the research conclusion that student interdisciplinary integration ability is closely related to interdisciplinary course participation, or that students involved in interdisciplinary learning have better interdisciplinary integration ability than students studying a single discipline (Y.-Y. Li and Lin 2018; Newell 1992; Wright 1992). However, this finding is similar to the findings of the 2017 study by Lattuca et al. Their research shows that students of interdisciplinary learning are not necessarily better than students of monodisciplinary learning in interdisciplinary-related abilities, and students’ acquisition of the ability may not necessarily change significantly due to the interdisciplinary characteristics (Lattuca et al. 2017b). Lattuca et al. also pointed out that their findings are consistent with evidence from Jacobs’ analysis of Arum et al.’s data (Lattuca et al. 2017b).

What causes this insignificant difference? Soares believed that curriculum designers often seem to underestimate the support that students need in interdisciplinary learning (Soares et al. 2013). Borrggo et al. suggested that the design of course projects should avoid as much as possible the frustration of students due to overly difficult problem tasks (Borrego et al. 2013). In fact, the research of Soares, Borrego et al. confirmed the systematic relation between interdisciplinary teaching and learning summarized by Spelt et al. through literature review, i.e., the impact of student attributes and interdisciplinary learning environment on interdisciplinary integration ability (Spelt et al. 2009). This does echo our findings. The results of the quantitative analyses and the feedback of learning conditions in the qualitative analyses should reflect the level of student interdisciplinary integration ability. In the qualitative analyses of these interviews, student attributes, including motivation, prior experience, and individual traits, and the interdisciplinary learning environment, including pressure and burden, and disciplinary factors, should be the influencing factors of student interdisciplinary integration ability. Meanwhile, from the analyses of the frequency of mentions in the coding analysis research, student attributes’ impact on interdisciplinary learning is significantly more than the interdisciplinary learning environment; and for each category, their influence from more to less is respectively: Motivation, Prior Experience, Individual Traits, Pressure and Burden, and Disciplinary Factors. We will further explore these five categories further below.

Motivation

In our interviews, the teachers pointed out that the students are unwilling to participate in the interdisciplinary program and dawdle their time away during interdisciplinary learning due to their lack of identification with interdisciplinary learning; students may join the program for some other reasons, so they are not very active; students think that they have spent time on interdisciplinary learning, but it does not help achieve their short-term goals, so they naturally withdraw from the program; students feel that they have spent energy on interdisciplinary learning, but the results are not satisfactory, and they are worried that their average score will be lowered, so they have negative reactions. Clearly, these are manifestations of a lack of motivation in interdisciplinary learning (see Appendix D for details). Motivation is defined as the process of initiating and maintaining goal-directed activities, while the goal-directed theory states that learners tend to engage in tasks related to mastering content or to do better than others or to avoid failure (Cook and Artino 2016). In addition, as an important part of motivational structure, self-efficacy (Lishinski et al. 2016) determines how much effort people are willing to put in, as well as people’s ability to cope and persevere in the face of challenges and difficulties (Bandura 1977). The discussion of motivation by Cook, Lishinski, and Bandura et al. should be sufficient to explain the impact of students’ lack of motivation for interdisciplinary learning. Therefore, whether from the frequency results of the qualitative analyses or from previous research on learning motivation, perhaps the primary task of interdisciplinary education should be the cultivation, shaping, and enhancement of learning motivation.

How can we shape or enhance student motivation for interdisciplinary learning? In the interviews, some teachers mentioned that students may need to know what kind of ability the interdisciplinary program is designed to cultivate, or what they may get after completing the program, which may be important motivation to support them to continue their studies. In this regard, some researchers pointed out that understanding the utility and importance of interdisciplinary learning is very important for student interdisciplinary learning outcomes (Chen et al. 2009; Matthews et al. 2010). In addition, Keller pointed out that establishing students’ motivation to learn requires successfully establishing the relevance of teaching to students as an individual (Keller 1987). In fact, the respondents reported that they have conveyed under the interdisciplinary program to the students the idea that interdisciplinary learning is more conducive to acquiring the ability and vision of innovation and entrepreneurship, but this does not seem to be related to students’ more realistic short-term goals of furthering their study, going abroad, finding a job, or improving their average score of their own discipline, so they fail to convince these students to realize the importance of interdisciplinarity. Obviously, this relatively superficial interdisciplinary concept transfer has not successfully established the relevance of teaching to students. This may be one of the reasons for not effectively stimulating students’ motivation for interdisciplinary learning. This is similar to the findings of Self et al.’s 2019 study, i.e., compared with British students, Korean students cannot be identified with the interdisciplinary nature of a particular occupation, and they are particularly concerned about the appropriateness of interdisciplinary education in terms of employment, its negative impact on employment, and are worried about whether interdisciplinary education will be valued by discipline-oriented industries. Self et al. believed that different regional cultures may influence students’ driving force of interdisciplinary learning (Self et al. 2019). Based on this, we infer that the students in East Asia may be more concerned about the relevance of interdisciplinary learning and the realization of short-term goals. In interdisciplinary education, the shaping or enhancement of student learning motivation should focus on this. In addition, judging from the introduction to the course teaching mentioned in the second part of this paper, for the students from different disciplines, the interdisciplinary basic courses under this program still use the original traditional teaching methods of each college. We speculate that this is bound to make it difficult for the students to establish the relevance of their own disciplines and interdisciplinary course teaching. Meanwhile, the original teaching methods of various disciplines retained in the teaching of interdisciplinary basic introductory courses have turned the teaching of interdisciplinary basic courses into multi-disciplinary teaching of disciplines plus disciplines, and fail to promote the integration of knowledge, methods, and viewpoints of various disciplines, which may make it difficult for the students from different disciplines to have effective interdisciplinary communication and interdisciplinary teamwork under this program. As Keller once pointed out, students’ effective learning and expectations of success are hindered, which will also lead to a decrease in learning motivation (Keller 1987). Therefore, although this program has interdisciplinary integration courses in the later stage, it still uses the traditional teaching methods in the interdisciplinary basic introductory courses in the early stage, which should also be the reasons that lead to the lack of students’ motivation and the hinderance of the improvement of students’ interdisciplinary integration ability.

Prior Experience

The courses students have taken can significantly influence their learning experience (Chen et al. 2009). The experience may affect student interdisciplinary learning. Spelt et al. pointed out that past social and educational experience, such as students’ previous thinking styles, the teaching styles they have been exposed to, and beliefs about the nature of knowledge and learning, may impact their interdisciplinary integration ability and thinking (Spelt et al. 2009). In our interviews, some teachers mentioned that students are not used to the teaching methods of the interdisciplinary teachers; if the students do not start to get used to the teaching methods in their freshman year, it will be hard for them to be adapted to them in their junior year; the engineering students cannot adapt to problem-oriented learning in basic design courses, and cannot understand teaching methods that do not have the best solution to problems in integrated courses; the students have no successful experience in innovation and entrepreneurship, so it will be difficult for them to understand and identify with the teachers’ perspectives on interdisciplinary learning(see Appendix D for details). In contrast, design students, as mentioned above, have more opportunities to be exposed to interdisciplinary knowledge, have earlier problem-oriented learning experiences, and are more adaptable to interdisciplinary learning. Meanwhile, judging from the quantitative results of students’ ability, non-interdisciplinary design students have significant performance in Communication 5, Reflection 1, and Practice 4 in the questions can also explain this. The information gathered supports Spelt et al.’s perspective. Based on this, the authors infer that students may experience discomfort or confusion in new learning due to differences in their previous study habits or teachers’ teaching styles. Meanwhile, the lack of specific interdisciplinary experience will lead to students’ failure in interdisciplinary value formation, which is not conducive to the construction of interdisciplinary learning motivation and the improvement of interdisciplinary integration ability level. This is consistent with Ramalingam et al.’s point that student self-efficacy and academic performance are positively related to their prior experience (Ramalingam et al. 2004), i.e., the amount of prior experience affects the amount of student’s interdisciplinary learning motivation and how much the learning effectiveness will be improved. On the other hand, as far as learning is concerned, researchers in the field of cognitive theory believed that how new information is organized and interrelated with previous knowledge has an important impact on learning, and interdisciplinary teachers should help students create a clear link between what they are going to learn and their prior experience, including what they have learned in the past (Lattuca et al. 2004). We obtained similar confirmation from the discussion of motivation in the previous part of this paper. In practice, however, it is not an easy task for interdisciplinary teachers to correlate students’ experience before and after interdisciplinary learning. It can be seen from the interviews that it is especially difficult for interdisciplinary teaching practitioners to understand and organize students’ prior non-educational experience. Therefore, interdisciplinary education should be regarded as a long-term process, and students should be exposed to interdisciplinary learning earlier to have interdisciplinary experience, which may gradually build students’ interdisciplinary cognition, establish their interdisciplinary values, facilitate the growth of interdisciplinary learning motivation, and promote the improvement of interdisciplinary integration ability. In this regard, Wilhelmsson et al. have the same understanding: the acquisition of interdisciplinary integration ability is a process that must start early in education (Wilhelmsson et al. 2009).

Individual Traits

The results of the interview analyses of this category show that the students majoring in Engineering, Management, and Design have different focuses and ways of dealing with problems in interdisciplinary learning, and the impact of their learning attitudes on learning outcomes. Several teachers pointed out that in interdisciplinary learning, Engineering students seem to be more conservative, so they think in a less creative way; Design students are original and have many ideas, but their consideration of practical application may be incomprehensive; Business students intend to save effort in their learning, and they often avoid wasting energy, time, and other learning risks. In this regard, some teachers pointed out that it is easier for the students to believe the value that is easier for them to understand or is more similar to their own major. On the other hand, the teachers believed that a serious attitude, a sense of responsibility and self-discipline reflected in students’ individual traits are still important factors for positive outcomes in interdisciplinary learning. Especially students who are willing to use what they have learned to analyze and organize can be a high achiever in the end (see Appendix D for details). It may be inferred that the values or learning styles of different disciplines affect students’ perspectives on problems and the learning strategies and actions they take. Meanwhile, students’ individual traits also seem to affect their own learning strategies, and thus affect the final interdisciplinary learning outcomes. Some researchers believed that disciplines affect the learning methods students adopt over time (Tarabashkina and Lietz 2011). The study by Bruce et al. found that for successful interdisciplinary learning, personalities and attitudes should be at least as important as disciplinary foundations and specialization. They believed that an excellent interdisciplinary person should have a high tolerance for ambiguity, and they should not prematurely narrow a problem to a limited set of dimensions, but instead, they should spend time exploring a range of dimensions and testing several potential boundaries; therefore, they also believed that an ideal interdisciplinary person should have curiosity about and willingness to learn other disciplines, and be open to the ideas and experience from other disciplines, etc. (Bruce et al. 2004). In this regard, Woods also believed that curiosity and openness represent a willingness to suspend doubts about other disciplinary cultures and suspend a hold on beliefs in their own disciplinary culture (Woods 2007). Tik believed that openness refers to the characteristics of students who are curious and intelligent. They are open to new experiences and willing to adopt other learning strategies; while responsibility refers to the characteristics of achievement, organization and perseverance, and students with these traits tend to be more inclined to use higher-order cognitive skills, such as critical thinking and metacognition (Tik 2020). Therefore, both students’ own individual traits and their characteristics caused by discipline attributes should have an impact on their interdisciplinary learning outcomes. Meanwhile, as shown in Table 7, the influence of students’ individual traits is greater than students’ characteristics caused by discipline attributes based on the number of respondents who mentioned them and the frequencies of mentions. Together with the previous research on student learning attitudes, this may show that the influence of student individual traits is slightly more important than the influence of student discipline characteristics on interdisciplinary learning outcomes.

Pressure and Burden

The interview materials of this category reflect that the intensity of learning and the increased strictness of teachers’ demands for task completion appear to lead to negative effects on student interdisciplinary learning. For example, the teachers pointed out that the students reported that the courses in the department of Design bring a heavy course load, and the courses given by the teachers of the department of Engineering are too in-depth, so that the students feel a heavy burden; in the later integrated courses, the students cannot accept the course output requirements and strictness of the teachers. In addition, students’ inadaptation of interdisciplinary learning also causes them to worry that their academic GPA will be lowered, so they think interdisciplinary learning is a burden for them, and eventually many students withdraw from the program (see Appendix C for details). This finding may be supported by Matthews et al. They embed programming teaching content in the study of first-year Biology undergraduates and required students to apply their programming skills in a quantitative real-world setting. However, it was too complex for the students to respond effectively, so the students’ feedback on this were negative to a large extent (Matthews et al. 2010). Moreover, Chen et al. have similar findings. They pointed out that a heavy study load increases the difficulty of students participating in various courses outside their own discipline and reduces their attention paid to interdisciplinary learning. Meanwhile, this may be a reason for the declining trend of students’ interest and value in interdisciplinary learning (Chen et al. 2009). Indeed, judging from the total credits of undergraduate majors in the three colleges of the school, each major has 150 credits, and participating in this interdisciplinary program will add 35 credits, which is equivalent to adding more than four credits per semester and 70 credit hours of lessons. In fact, the number of all the courses is not evenly distributed in each semester. If the interdisciplinary teachers have higher requirements on coursework and put more pressure on their students, especially in certain semesters with more class hours, students will definitely feel the weight of a heavier study load, and as a result, they will naturally choose to give up interdisciplinary learning to ensure their own disciplinary learning. Therefore, when designing interdisciplinary curriculum content and student output requirements, teachers should comprehensively consider the pressure and burden brought to students by the learning load of both interdisciplinary courses and the courses of their own discipline. This requires more adequate and effective communication and coordination between interdisciplinary teachers and teachers of different disciplines, in order to bring positive effects on student learning outcomes.

Disciplinary Factors

Teachers’ disciplinary views and biases can influence how students learn and experience in interdisciplinary learning (Self et al. 2019). Self et al. found that some teachers’ own disciplinary biases can be transformed into their expectations for students, which leads students to change their learning methods and learning outcomes in their studies to meet the expectations of disciplinary teachers. Our research findings support this view. The interview participants indicated that disciplinary teachers are accustomed to using their values to influence students. They lack support for the students participating in interdisciplinary courses. For example, non-interdisciplinary teachers show their inhibition or contempt for interdisciplinarity or the interdisciplinary program before the students being involved in interdisciplinary leaning when teaching their own disciplinary courses, so that the students have distrust of interdisciplinary courses and teachers. In fact, this attitude of disciplinary teachers towards interdisciplinary learning should be relatively common. First, teachers who lack interdisciplinary experience may also lack enthusiasm or willingness to develop interdisciplinary projects (Gardner et al. 2014; Van den Beemt et al. 2020). Second, the academic community and higher education community generally regard disciplines as cornerstones, so they tend to marginalize more comprehensive areas of knowledge or educational programs (Palaiologou 2010). Brew suggested in his research that many scholars tend to overemphasize the importance of disciplinary affiliation (Brew 2008). In this regard, Lindvig et al. believed that interdisciplinary teaching, which is different from the accustomed way of disciplinary teaching, may be regarded as a threat to hinder the construction of the disciplinary identity, so this should be one of the difficulties that interdisciplinary teaching is facing (Lindvig and Ulriksen 2019). Obviously, the influence of teachers’ words and deeds based on disciplinary thinking and values brings challenges to students in interdisciplinary learning. In the operation and management of interdisciplinary programs, schools need to establish common interdisciplinary educational values among teachers of various disciplines to avoid negative impact on interdisciplinary teaching by disciplinary teachers who are not involved in interdisciplinary teaching. In addition, some respondents pointed out that Design teachers and Engineering teachers have different evaluation criteria for student outputs, which has led to students’ frustration in interdisciplinary learning. For example, Engineering students’ award-winning works in disciplinary competitions cannot be recognized by Design teachers (see Appendix C for details). This is consistent with the findings of Self et al., and in their study, professors of Industrial Design rarely collaborate with professors of Ergonomics, and the differences between these two disciplines have an impact on course learning outcomes. What is considered important by everyone is not considered important in Ergonomics (Self et al. 2019). If such disparities between disciplines are not balanced and integrated to form judging criteria based on a shared value, challenges will be created for interdisciplinary learning and teaching.

## 5. Conclusions

This study uses the Core Interdisciplinary Integration Ability Scale developed by Chen et al. to measure the interdisciplinary integration ability of the students participating in the interdisciplinary program that integrates design, engineering, business and other disciplines. Under the theoretical framework of Biggs, Spelt, and Liu et al. on interdisciplinary learning outcomes, student attributes, and interdisciplinary learning environment, a qualitative analysis of interviews with interdisciplinary teachers and related personnel is conducted. The research group found that there is no significant difference in interdisciplinary integration ability between the students participating in the interdisciplinary program and the students involved in a single discipline study, including the Industrial Design, Electrical Engineering, and Materials Engineering students. Based on the qualitative analysis results of the interview data, the authors believe that the reasons why there is no significant difference may be problems with student attributes, including the lack of motivation, lack of prior interdisciplinary experience, influence of individual traits, and problems with interdisciplinary learning environment, including the increased learning pressure and burden, and interference of disciplinary factors. Our findings can provide some references for the future development and design of interdisciplinary programs and interdisciplinary teaching. Especially for the establishment and shaping of interdisciplinary learning motivation, for students in East Asia, attention should be paid to the substantial connection between students’ short-term goals and interdisciplinary learning, as well as to the construction of the correlation between students’ own disciplines and interdisciplinary learning content; meanwhile, for the interdisciplinary basic course teaching in the early stage of interdisciplinary programs, we should take into account the fact that the students under these programs are from different disciplines, and carry out teaching from the perspective of knowledge integration, so as to avoid using original discipline teaching methods to simply make interdisciplinary teaching into multi-disciplinary teaching. Besides, it may be beneficial to start students’ experience in interdisciplinary learning or research at an earlier stage to gradually form students’ interdisciplinary cognitions and values. In addition, when establishing a teaching design for students, attention should be paid to their individual traits and there should be sufficient communication and coordination with students’ disciplinary teachers to achieve a balance between interdisciplinary and disciplinary learning, form a commonly recognized evaluation standard, and try to avoid negative effects on the learning outcomes of interdisciplinary students due to the increase of students’ learning pressure and academic burden or the interference of disciplinary factors.

Due to the different systems and structures of interdisciplinary programs among universities, this study did not collect data from other universities for comparison. Besides, because of some students’ unwillingness and the impact of the COVID-19 pandemic, the research group did not collect any samples of students majoring in Business Management and all tested students’ opinions on the directness of the interdisciplinary courses in this university. With the graduation of this group of students, the collection of relevant samples has become unlikely. The lack of such sample data makes it difficult for us to truly and directly understand the psychological state and opinions of the students participating in this interdisciplinary program. Only relying on the teachers’ observation, description and evaluation of the students may miss the details of some students’ conditions, resulting in some problems not being discovered in time. This indeed brings about some limitations to this research. Fortunately, the measurement results of students’ interdisciplinary ability and the analysis results of teacher interviews can confirm each other, so this research group believes that our experimental data are convincing. Our findings further confirm the previous view that students participating in interdisciplinary learning may not necessarily improve their interdisciplinary ability. Meanwhile, on this basis, according to the empirical results, this study points out the specific factors that bring interdisciplinary learning challenges to students. This provides inspiration for subsequent related research. In addition, this study only discusses the impact of student attributes and interdisciplinary learning environment on learning outcomes, but from the theoretical model of Biggs et al., learning outcomes can also affect student attributes and learning environment. This will open the way for our future research considering, e.g., how the improvement of students’ interdisciplinary ability will stimulate students’ interdisciplinary motivation.

## Figures and Tables

**Table 1:** The Above-Mentioned Core Interdisciplinary Integration Abilities.

Core Interdisciplinary Abilities	Scholar/Institution			
	IPEC	Wilhelmsson et al.	University of Virginia	Lattuca et al.
Interdisciplinary Communication	x	x	x	
Interdisciplinary Teamwork /Shared Problem-Solving /Shared Decision Making /Shared Knowledge or General Common Knowledge Base/Conflict Resolution	x	x	x	x
Reflection/Interdisciplinary Evaluation		x		x
Appreciation of Non-Disciplinary Perspectives/Values/Ethics for Inter- Professional Practice	x	x		x
Recognition of Disciplinary Limitations/Awareness of Disciplinarity/Appreciation of Disciplinary Perspectives				x
Integrative Skill				x
Professionalism/Responsibilities	x		x	

**Table 2:** The Interdisciplinary Program.

Course Selection Semester	Course Name	Credit	Mutually-Recognized Course	Note
1st sem.	Social Design	2	General Education Course (Social Science)	Public Compulsory Courses under the Interdisciplinary Program
	Makeathon	2	General Education Course (Humanities and Arts)	
2nd sem.	Introduction to Computational Thinking and Data Science	2	General Education Course (Natural Science)	
	English Communication	2	General Education Course (General Knowledge of Language)	
3rd–5th sem.	Economics that Can Be Seen Everywhere	1	Free Electives Credits for Mechanical Engineering, Electrical Engineering, Computer Science and Engineering, and Design Majors	Compulsory Courses for Design and Engineering Majors
	Financial Statements	1		
	Financial Economics	1		
	Business Analysis: Costs and Decisions	1		
	Capstone for Management	2	General Education Course (Social Science)	
	Creation Processing	1	Free Electives Credits for Management and Design Majors	Compulsory Courses for Management and Design Majors
	Applied Electronic Creation	1		
	Institutional Design Practice	1		
	Materials Processing and Analysis	1		
	Capstone for Engineering	2	General Education Course (Natural Science)	
	Design Expression Methods	2	Free Electives Credits for Mechanical Engineering, Electrical Engineering, Computer Science and Engineering, and Management Majors	Compulsory Courses for Management and Engineering Majors
	Design Fundamentals	1		
	Colorful Material Surface Treatment	1		
	Capstone for Design	2	General Education Course (Humanities and Arts)	
	Off-Campus Internship	2	Off-Campus Internship for Different Majors or Free Elective Credits	Elective 2 Credits
	Exchange Abroad	2		
6th sem.	Thematic Interdisciplinary Course I	3	Thematic Courses for Different Majors or Free Elective Credits	Public Compulsory Courses under the Interdisciplinary Program
7th sem.	Thematic Interdisciplinary Course II	3		
8th sem.	Innovation and Entrepreneurship	2		

**Table 3:** Measurement Variables and Number of Students Tested.

Participation in the Interdisciplinary Program	Number of Students Tested	Independent Variables	Dependent Variables
Yes	19	Interdisciplinary Students	Interdisciplinary Communication
No	23	Industrial Design Students	
			Interdisciplinary Reflection
No	29	Materials Engineering Students	
			Interdisciplinary Practice
No	20	Electrical Engineering Students	

**Table 4:** Results of the One-Way ANOVA.

Dependent Variables	d.f.	*F*	Sig.
Communication 2	3, 87	2.852	.042
Communication 5	3, 87	3.932	.011
Reflection 1	3, 87	3.035	.033
Reflection 3	3, 87	2.769	.046
Practice 1	3, 87	2.751	.047

**Table 5:** Results of the Test of Homogeneity of Variance.

Dependent Variables	Levene Statistic	d.f.	Sig.
Communication 3	3.011	3, 87	.034
Reflection 4	5.925	3, 87	.001
Practice 2	3.513	3, 87	.019
Practice 4	3.416	3, 87	.021

**Table 6:** Results of the Post Hoc Tests: Multiple Comparisons.

Dependent Variables	Comparison Method	(I) Independent Variables	(J) Independent Variables	(I–J) Mean Difference	Std. Error	Sig.
Communication 5	Scheffe	EE	ID	−.67391 *	.22172	.032
			ME	−.63793 *	.21078	.033
			InterD	−.50000	.23232	.209
Reflection 1	Scheffe	ID	ME	.39280	.17829	.191
			EE	.56522 *	.19523	.045
			InterD	.30206	.19796	.510
Practice 4	Games-Howell	ID	ME	.15592	.16461	.780
			EE	.48696 *	.15955	.022
			InterD	.08696	.17594	.960

* represents Sig. < .05, InterD = Interdisciplinary, EE = Electrical Engineering, ID = Industrial Design, ME = Mechanical Engineering.

**Table 7:** Codes and Frequencies.

Encoding Dimension	Encoding Category	Encoding Subcategory	Number of Respondents	Frequency of Mentions
D-01 Learning Condition Feedback	D-01-01 Positive Feedback		2	8
		D-01-01a Growth in Interdisciplinary Ability	2	5
		D-01-01b Other Positive Feedback	1	3
	D-01-02 Negative Feedback		6	28
		D-01-02a Problems with Interdisciplinary Ability	5	18
		D-01-02b Student Distrust	2	5
		D-01-02c Negative Emotions and Behaviors	3	5
D-02 Interdisciplinary Learning Environment	D-02-01 Pressure and Burden		6	19
		D-02-01a Study Pressure	4	8
		D-02-01b Academic Burden	6	11
	D-02-02 Disciplinary Factors		6	15
		D-02-02a Influence of Departments	3	10
		D-02-02b Difference among Disciplines	5	5
	D-02-03 Social Support		1	7
		D-02-03a Lack of Family Support	1	5
		D-02-03b Lack of Other Social Support	1	2
D-03 Student Attributes	D-03-01 Motivation		8	56
		D-03-01a Intrinsic Motivation	8	38
		D-03-01b Extrinsic Motivation	2	9
		D-03-01c Source of Motivation	6	9
	D-03-02 Prior Experience		7	29
		D-03-02a Influence of Prior Teaching and Learning Styles	6	21
		D-03-02b Prior Interdisciplinary Practice Experience and Cognition	3	8
	D-03-03 Individual Traits		5	23
		D-03-03a Different Characteristics of Students in Different Departments	3	10
		D-03-03b Student Personal Characteristics	5	13

**Table 8:** Frequency Proportion and Ranking of Each Dimension and Category Mentioned.

Encoding Dimension	Encoding Category	Frequency Proportion within Main Axis Dimension	Total Frequency Proportion	Ranking of Frequency of Main Axis Dimension	Ranking of Frequency of Category
D-01 Learning Condition Feedback		100%	19.4%	3	
	D-01-01 Positive Feedback	22.2%	4.3%		7
	D-01-02 Negative Feedback	77.8%	15.1%		3
D-02 Interdisciplinary Learning Environment		100%	22.2%	2	
	D-02-01 Pressure and Burden	46.3%	10.3%		5
	D-02-02 Disciplinary Factors	36.6%	8.1%		6
D-03 Student Attributes		100%	58.4%	1	
	D-03-01 Motivation	51.9%	30.3%		1
	D-03-02 Prior Experience	26.9%	15.7%		2
	D-03-03 Individual Traits	21.3%	12.4%		4

## Data Availability

The data presented in this study are available on request from the corresponding author. The data are not publicly available due to The relevant data involves the internal information of the institution and personal information, and there is a need for confidentiality.

## Appendix A

**Table A1:** Core Interdisciplinary Integration Ability Scale Questions.

Interdisciplinary Integration Sub-Ability	Scale Questions
Interdisciplinary Communication	1. I can listen to professional opinions from students with different expertise.
	2. I can give feedback to students with different expertise.
	3. I can understand the main ideas being discussed when discussing with students with different expertise.
	4. I can understand the professional terms that students with different expertise use when communicating.
	5. I can use effective communication tools to facilitate communication with students with different expertise.
	6. I can use effective communication tools to promote consensus among students with different expertise.
Interdisciplinary Reflection	1. I can understand the reasons why students with different expertise have different opinions when working with them to complete tasks.
	2. I can reflect on my own opinions from interactions with other students when working with them to complete tasks.
	3. I can generate new ideas from interactions with other students when working with them to complete tasks.
	4. I can clarify the current problems encountered in the process of completing tasks when working with other students to complete tasks.
	5. I can actively seek solutions to possible problems encountered when working with other students to complete tasks.
Interdisciplinary Practice	1. I can propose practical solutions to problems identified in the process of completing group tasks.
	2. I can assess my own performance in a group when working with my groupmates to complete tasks.
	3. I can assess the performance of my groupmates when working with them to complete tasks.
	4. I can assess the overall results achieved by my group after working with my groupmates to complete tasks.
	5. I can make specific suggestions for improving the results achieved by my group after working with my groupmates to complete tasks.

Compiled and translated from (Chen et al. 2017).

## Appendix B

**Table A2:** Coding of D-01 Learning Condition Feedback.

Category	Respondent Code	Encoded Text
D-01-01	Pt-8:	After all, they are from different departments, so uh … it may be a little difficult for them to exchange opinions. But in the end, they can discuss a way that everyone may accept.
	Pt-8:	So I think when it comes to feedback, you can see that, uh … their ability increases … and they reach a consensus, and they really agree with what they have discussed.
	Pt-9:	There is one Materials student, and it is easy for him … He was the first to form a group … He formed a group with three students from the department of Industrial Design in no time.
	Pt-9:	They are happy that they are in a group now.
D-01-02	Pt-1:	In fact, I do not think they (the students) have identification with this (interdisciplinary learning).
	Pt-2:	Last time, a student of Industrial Design came to ask me a question … He wanted to connect to Ubike (the name of a shared bicycle in Taipei) via Bluetooth, and I told him, ‘You should use NFC instead of Bluetooth. The bicycle will be unlocked at a distance of 30 to 40 cm when being connected via Bluetooth. If someone just stands next to your bicycle when it is unlocked and he (/she) rides it away, what could you do?’ He did not understand or accept what I said. In fact, Design teachers and Engineering students also communicate in this way …
	Pt-7:	One (Engineering) student said that he was involved in wafer manufacturing process, and then a (Design) teacher questioned him, ‘What is your purpose in doing this? What is the point of making something that is already available on the market? … You have to consider what your role is and what your contribution is in this whole process, as well as what your contribution to society is, and what the final product is. It is very strange that an Engineering student does not care about these things. You just focus on a small part of manufacturing process.’ After hearing this, the Engineering student was so angry and he really doubted whether these problems existed.
	Pt-7:	The students tend to believe in the value that is easier for them to accept, and then they would use their own value to challenge what we want to pass on them …
	Pt-1:	… They have talked about this for a month, and still cannot understand each other … Every time when there is a discussion, I have to join them to make an interpretation. For example, I must interpret what the Electrical Engineering students have to say for the students from the department of Design … I really wonder whether these students can understand each other.
	Pt-1:	The students have been questioning why we can guide them since we do not seem very professional.
	Pt-7:	The students do not have much trust in interdisciplinarity. This is what we have observed in PBL(Problem-Based Learning).
	Pt-7:	And they (some students) said that, ‘The teacher is treating us as white mice in terms of teaching design.’ It means that they think the teacher does not take the course seriously either …
	Pt-8:	Of course there are times when these students are a little … uh … listless or less willing to engage in discussions.
	Pt-9:	When the teachers have high requirements or the courses are far from the expectations of the students, it would be easy for them to give up.
	Pt-7:	You can see a lot of, uh … the students’ frustration and disputes with peers, and then they just disappeared.
	Pt-7:	I gave him a score of 75, and was scolded by him. He said angrily, ‘How could you not care about whether your students are applying for Learning Excellence Awards, or whether they are planning to apply for schools abroad in the future?’

Under the principle of not changing the meaning of the respondents’ conversations, in order to show the content of the conversations more accurately, the authors annotate what has been omitted or referred to in the conversations through ( ) according to the context of the interviews.

## Appendix C

**Table A3:** Coding of D-02 interdisciplinary Learning Environment.

Category	Respondent Code	Encoded Text
D-02-01	Pt-3:	So when Engineering students are collaborating with Design students, sometimes it can really be … The students cannot accept it when the teachers are strict with their task completion.
	Pt-1:	Previously I talked with two groups of students and they have never come back to me again. Instead, they turned to another teacher to sob out their misfortune. I think it was probably because I gave them too much pressure. Those two groups of students have never appeared in front of me since then.
	Pt-7:	When the students feel pressure, their response can be emotional and external … They may cry or curse when they respond to pressure.
	Pt-9:	I have heard some complaints from the students, which was either that the study load of the courses of Industrial Design is heavy or that their courses of Engineering are too difficult. Under these circumstances, they would feel frustrated and give up in the middle of their studies. When the students from the Department of Engineering, Business, Electrical Engineering, or Computer Science and Engineering attend the courses from the Department of Industrial Design, they often feel great pressure and it is also true to Design students taking Engineering courses.
	Pt-8:	Some students are concerned about their scores of these interdisciplinary courses because they plan to apply for graduate programs either in Engineering or Business Management in the future … So they can be anxious if they get low scores.
	Pt-1:	Many students would wonder what the point of taking these interdisciplinary courses in the first place is … Once their scores are not satisfying, which can lead to their lowered scores of their own discipline, and even failure in scholarship application, they would be unwilling to continue their study in these courses.
	Pt-7:	Once they believe that taking these courses consume too much energy, there is not much the teachers can do to help them complete their studies. They would quit soon.
D-02-02	Pt-7:	Conversations between many teachers and their students are all about … Talking about this (interdisciplinary learning) based on their own value, the teachers tend to … mislead their students …
	Pt-7:	Many teachers would, uh … blame the students for taking these disciplinary courses without having excelled at their own major first. I guess most of them hold a disapproving attitude.
	Pt-2:	And the teachers of their own major would certainly want the students to wholeheartedly complete their tasks. They would not give a thought about interdisciplinary stuff.
	Pt-8:	For instance, as for drawing, a student may spend a lot of time drawing a line or something that is a piece of cake for any Design student. But in the end, his drawing may not be any better or even fail to meet the given standards. As a result, he (/she) would get low scores and feel frustrated.
	Pt-7:	In my opinion, that stuff the student had been working on is … a nightmare for exhibition. I almost passed out at first sight of it. However, it had already won four awards in an Engineering competition. As a result, the student refused to listen to my suggestions. He said that he did not want to change any part of it.
	Pt-2:	The students from the Departments of Electrical Engineering, and Computer Science and Engineering are like: OK, I have learned a new trick. It would be just perfect if I can imitate it and add some change. But the teachers in the Department of Design would ask these students why they do this when there is probably no such market need. And they (these students) could not accept it at all.

Under the principle of not changing the meaning of the respondents’ conversations, in order to show the content of the conversations more accurately, the authors annotate what has been omitted or referred to in the conversations through ( ) according to the context of the interviews.

## Appendix D

**Table A4:** Coding of D-03 Student Attributes.

Category	Respondent Code	Encoded Text
D-03-01	Pt-1:	Perhaps these students just want to dawdle their time away during interdisciplinary learning, and they do not care much about whether they can get a high score.
	Pt-1:	In fact, these students do not want to be involved in public affairs and thematic interdisciplinary courses because they do not see any point in doing it.
	Pt-9:	It seems to me that these students do not have … yep, an impulse for learning. They are satisfied with what they have already had and do not want to give new stuff a shot to discover their potential. They do not think like this.
	Pt-4:	I think we can set up some attainable and attractive goals for them. In the beginning, these student may enroll in this program due to some external incentives, but in the long run, we hope that they may set goals for themselves and go for them. We hope that the students can have an outlook of their future, which is crucial to develop their motivation. Otherwise, they do not know why they should work so hard to meet these demanding requirements.
	Pt-7:	As for motivation, the students may have strong motivation to study their own major, but little motivation for the interdisciplinary courses.
	Pt-8:	When a student spends a lot of time learning Engineering and does not have a good result, he (/she) would start to think what the point of doing all these is. He (/she) may also get worried that his (/her) low score would affect the application for a scholarship or graduate program …
	Pt-2:	It is natural for the students to give up studying interdisciplinary courses and only take courses of their major when they think learning these interdisciplinary courses is only a waste of time … and does no good to help them achieve their short-term goals.
	Pt-4:	I think what motivated these students to participate in this program in the first place is that they wanted to go abroad or participate in overseas internship programs. This could be their original motivation. I hoped that during interdisciplinary learning, their original motivation generated by such incentives could be shifted to intrinsic motivation because it seemed to me that they did not really know why they participated in this program in a short run. I have heard many teachers in other universities complain that their students take interdisciplinary courses without any intrinsic motivation.
	Pt-7:	Many students take these interdisciplinary courses for the accompanied benefits … The university will finance them to go abroad and offer them scholarships. All these perks draw them in.
	Pt-8:	They may not know or understand what ability they can acquire, what knowledge they can get after finishing these courses, or what these courses are designed for. Knowing this is actually important because it may give rise to their major motivation for further studies.
	Pt-9:	Students’ identification with teachers can be potential motivation for them to take the course seriously because they think they are related to the teachers.
	Pt-7:	We do not have much influence over the students. Whereas it is always a challenge for us to hold their attention … They are expecting us to talk about jobs or graduate program application while we are talking about innovative choices such as starting a business. I know this is a little bit distant for them. Rich students do not have motivation to earn big money and poor students do not have the guts to bet all they have on uncertainty. They would rather focus on the study of their own discipline. The fact is that no parents would want their children to take an innovative career path. So for most of the students, their motivation to study is to get a high grade of their own discipline.
D-03-02	Pt-9:	Since their first year of university, the Design students have been challenged … Their teachers have been questioned or challenged their imagination with really difficult questions or questions without a specific answer. In contrast, the Engineering students normally would not be given too difficult or challenging tasks. Different learning environment is the reason why they have different learning styles that they are accustomed to.
	Pt-2:	As the Industrial Design students have been challenged by their teachers since the first year of university, they are more resilient than their counterparts in other departments when faced with criticism. I am not saying that students in other departments are not resilient. They just are not used to this learning style.
	Pt-7:	Many of the Design students have seen some graduation exhibition as early as in their high school … It was at that moment they decided to take it as their major. That is why they always have a sense of mission unaccomplished on their mind.
	Pt-7:	Many of the students may not know much about PBL courses. They require the students to find an answer without being given any specific guidance. The students would feel really troubled especially when they are taking Capstone courses in the third year. In this process, the teachers would keep asking the students to … find the best solution. The students can be stressed out when they are told there is no standard answer. So I think the students should … get used to it since the first year. Otherwise many of them would be likely to get frustrated when the teachers tell them there is no one specific correct answer to the question in their third or fourth year.
	Pt-7:	When I tell the students that interdisciplinary learning can help them start a business, I think it is really difficult to persuade them because most of them do not have enough related experience.
	Pt-1:	Actually many students do not have a clear picture in the beginning, so they are not aware of what they are facing.
D-03-03	Pt-8:	I think … Engineering students may not be good at … communication. So I usually advise them … to improve their communicative skills for team projects. These students are also conservative and less creative while Industrial Design students are much more ingenious and have more new ideas. But I also noticed that Design students are more likely to have trouble putting their ideas into practice … due to their incomprehensive consideration.
	Pt-8:	Design students, uh … take esthetics as the top priority, and they think their ideas can only be demonstrated in a certain way while Engineering students think, uh … the cost is the most important.
	Pt-9:	It seems to me that … Industrial Design students have stronger learning ability … or more solid basic skills.
	Pt-7:	Business students are much more different. They prefer to work with the students of their own discipline, and talk like CEOs … Their personality is … How to put it … They tend to take shortcuts. They are taught to avoid risks … and save effort and energy.
	Pt-7:	Based on my experience, Engineering students are more willing to communicate with me even though they are in a mood. You can see that they are conservative and rational. And many of the Design students are willing to accept criticism.
	Pt-1:	I know three third-year Design students who are inquiring their teachers about the thematic interdisciplinary course in the fourth year. As far as I know, they are conscientious and capable of doing design projects. They have already had plans on how to carry out interdisciplinary projects in the future …
	Pt-7:	These students are rational … and capable of integrating and analyzing what they have learned … We can see that students who have good academic performance and successful careers have these qualities.
	Pt-7:	For example, some students may not be impressive in school or active in learning their own discipline. What they have achieved now, which may not be so ideal … reflects their individual traits.
	Pt-8:	As I mentioned before, my course is not that difficult, uh … as long as the students are willing to spare no efforts to study. I think their attitude towards learning is what counts.
	Pt-9:	With a sense of obligation, some students are willing to work hard though they would complain from time to time… Generally speaking, disciplined students always get good results as we expect.

Under the principle of not changing the meaning of the respondents’ conversations, in order to show the content of the conversations more accurately, the authors annotate what has been omitted or referred to in the conversations through ( ) according to the context of the interviews.
